# Supplementary material for: Short Chain Fatty Acid Biosynthesis in Microalgae Synechococcus sp. PCC 7942
Source: Mar Drugs. 2019 Apr 28;17(5):255. doi: 10.3390/md17050255 (PMC6562792; doi:10.3390/md17050255)
Supplement: Supplementary file 1 [file marinedrugs-17-00255-s001.pdf]

Supplementary Materials:

Table S1. Primers used in this experiment.

**Primer name**	**Sequence (5'to 3')**	**Purpose**
7942-anti-fabB/F-F	GGGTAACCCATGACTGAAACCGGACGCC	construction of
7942- anti -fabB/F-R	GGGGTACCCC CTAGGGATGGAATTTCCGGAAG	antisense
7942- anti -fabH-F	GGGTAACCCTTGACTCGACCTGGCGTTG	expression vector
7942- anti -fabH-R	GGGGTACCCCCTAAACCACCGTGCCCCAAC	for *fabB/F* (*H*) of *Synechococcus* sp. PCC 7942
7942-sen-fabB/F-F	GGGGTACCCCATGACTGAAACCGGACGCC	construction of
7942-sen-fabB/F-R	CCGCTCGAGCGGCTAGGGATGGAA TTTCCGGAAG	sense expression vector for *fabB/F*
7942-sen-fabH-F	GGGGTACCCCTTGACTCGACCTGGCGTTG	(*H*) of
7942-sen-fabH-R	CCGCTCGAGCGGCTAAACCACCGTGCCCCAAC	*Synechococcus*sp . PCC 7942
7942- fabB/F-qPCR-F	TGATGGAGGACCAGCAGA	
7942- fabB/F-qPCR-R	TTCACCCACCGCATTAGA	quantitative
7942- fabH-qPCR-F	GTGAACTACGGCAATACCTCC	RT-PCR
7942- fabH-qPCR-R	CCTGCTCCAAACCCTGAA	
rnpB-F	ACCAGACTTGCTGGGTAACG	
rnpB-R	TTACCGAGCCAACACCTCTC	
7942-△fabB/F-F1	CGGAATTC ATGACTGAAACCGGACGC	construction of
7942-△fabB/F-R1	TGCAGCGATCGGGGCCTTTT	expression vector
7942-△fabB/F-F2	AAAAGGCCCCGATCGCTGCA GGAGCTAAGGAAGCTAAAATG	for *fabB/F* deletion mutants
7942-△fabB/F-R2	TTATTTGTACAATTCATCCATACCA	of
7942-△fabB/F-F3	GTCTTCAAATTCCCGTTGC GGCGATCGAGTTGGCGCTCC	*Synechococcus* sp. PCC 7942
7942-△fabB/F-R3	CCCAAGCTT CTAGGGATGGAATTTCCGGA	
7942-△fabB/F-F1	CGGAATTC TTGACTCGACCTGGCGTTGGCGT	construction of
7942-△fabH-R1	TTGCACTTGGCAGGCACTGC	expression vector
7942-△fabH-F2	GCAGTGCCTGCCAAGTGCAA GGAGCTAAGGAAGCTAAAATG	for *fabH* deletion mutants of
7942-△fabH-R2	TTATTTGTACAATTCATCCATACCA	*Synechococcus*
7942-△fabH-F3	GTCTTCAAATTCCCGTTGC ATCCTCGAGAAGACGCTGTTCC	sp. PCC 7942
7942-△fabH-R3	CCCAAGCTT CTAAACCACCGTGCCCCAAC	
7942-fabB/F-egfp-F1	TTATTTGTACAATTCATCCATACCA	construction of
7942-fabB/F-egfp-R 1	GGGATGGAATTTCCGGAAGG	expression vector for fluorescence

7942-fabB/F-egfp-F2	CCTTCCGGAAATTCCATCCC	localization
	TCTAAAGGTGAAG AATTATTCACTGG	
7942-fabB/F-egfp-F2	TTATTTGTACAATTCATCCATACCA	
7942-fabH-egfp-F1	TTGACTCGACCTGGCGTTG	construction of
7942-fabH-egfp-R1	AACCACCGTGCCCCAAC	expression vector
7942-fabH-egfp-F2	GTTGGGGCACGGTGGTT TCT AAAGGTGAAG	for fluorescence
	AATTATTCAC	localization
7942-fabH-egfp-F2	TTATTTGTACAATTCATCCATACCA	
ec-fabB-F1	CGGAATTCATGAAACGTGCAGTGATTACTG	construction of
ec-fabB-R1	CCACATACGGGCCAACCGCTTT	expression vector
ec-fabB-cat-F2	AAAGCGGTTGGCCCGTATGTGGGG	for *fabB* deletion
	AGCTAAGGAAGCTAAAATG	mutants of *E.coli*
ec-fabB-cat-R2	GCAACGGGAATTTGAAGAC	
ec-fabB-F3	GTCTTCAAATTCCCGTTGCCGAAGGCG	
	CAGTACGCTGCA	
ec-fabB-R3	CCCAAGCTT TTAATCTTTCAGCTTGCGC	
ec-fabF-F1	CGGAATTCGTGTCTAAGCGTCGTGTAGT	construction of
ec-fabF-R1	AGAATGGGCTGATCTTACGT	expression vector
ec-fabF-cat-F2	ACGTAAGATCAGCCCATTCTGGAGCTAA	for *fabF* deletion
	GGAAGCTAAAATG	mutants of *E.coli*
ec-fabF-cat-R2	GCAACGGGAATTTGAAGAC	
ec-fabF-F3	GTCTTCAAATTCCCGTTGCTCTGGC	
	AATGGCAAATGCTC	
ec-fabF-R3	CCCAAGCTT	
	TTAGATCTTTTTAAAGATCAAAGAA	
ec-fabH-F1	CGGAATT ATGTATACGAAGATTATTGGTACTG	construction of
ec-fabH-R1	CGGAATTC AATGCCCAGCATGCTTTGAA	expression vector
ec-fabH-cat-F2	TTCAAAGCATGCTGGGCATT	for *fabH* deletion
	GGAGCTAAGGAAGCTAAAATG	mutants of *E.coli*
ec-fabH-cat-R2	GCAACGGGAATTTGAAGAC	
ec-fabH-F3	GTCTTCAAATTCCCGTTGC	
	GAACTGGCGCACATCGTTGA	
ec-fabH-R3	CCCAAGCTT CTAGAAACGAACCAGCGC	

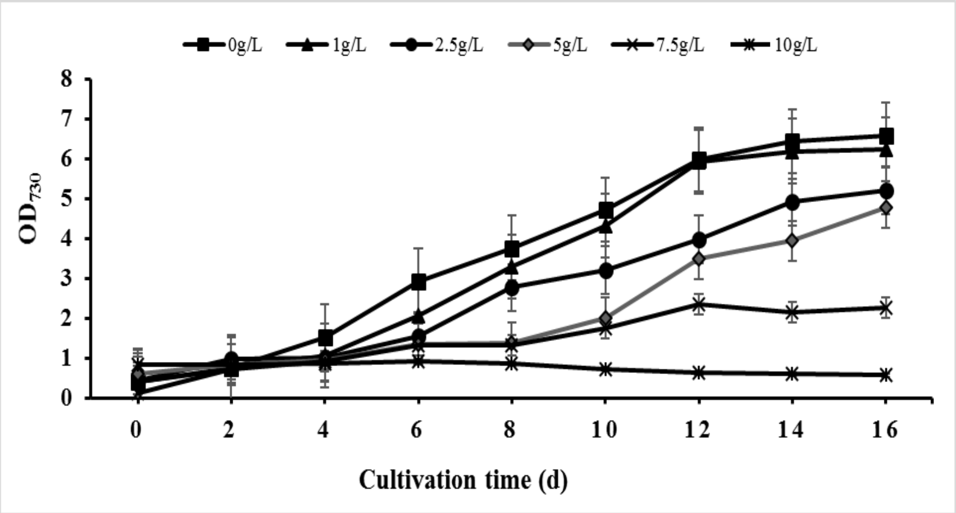

Figure S1. Effects of different cerulenin concentrations on the growth of *Synechococcus* sp. PCC 7942

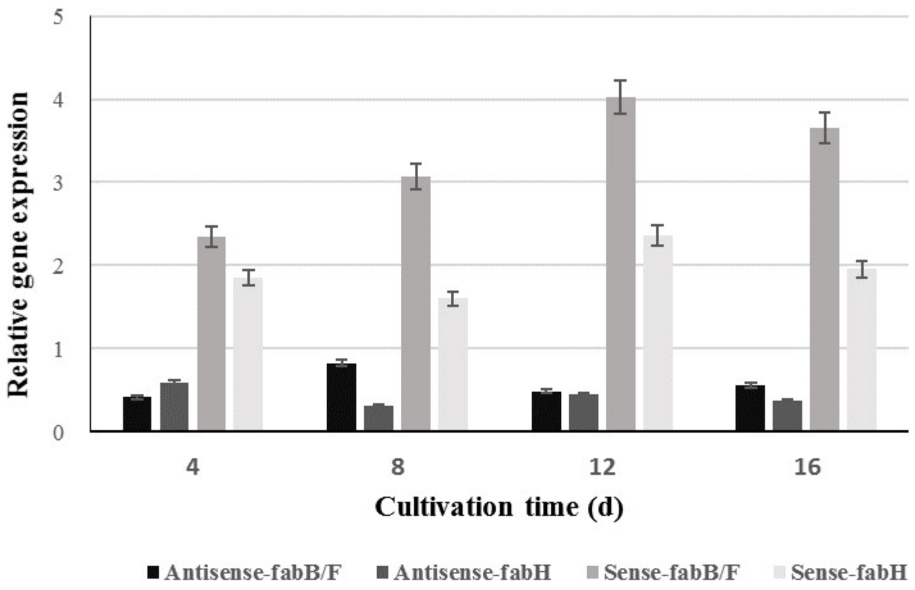

Figure S2. The expression levels of *fabB/F* and *fabH* in *Synechococcus* sp. PCC 7942 with sense and antisense expression of Synpccw7942_0537 (*fabB/F*) and Synpccw7942_1455 (*fabH*). The expression levels of Synpccw7942_0537 (*fabB/F*) and Synpccw7942_1455 (*fabH*) in wild type strain were set to 1.

(a)

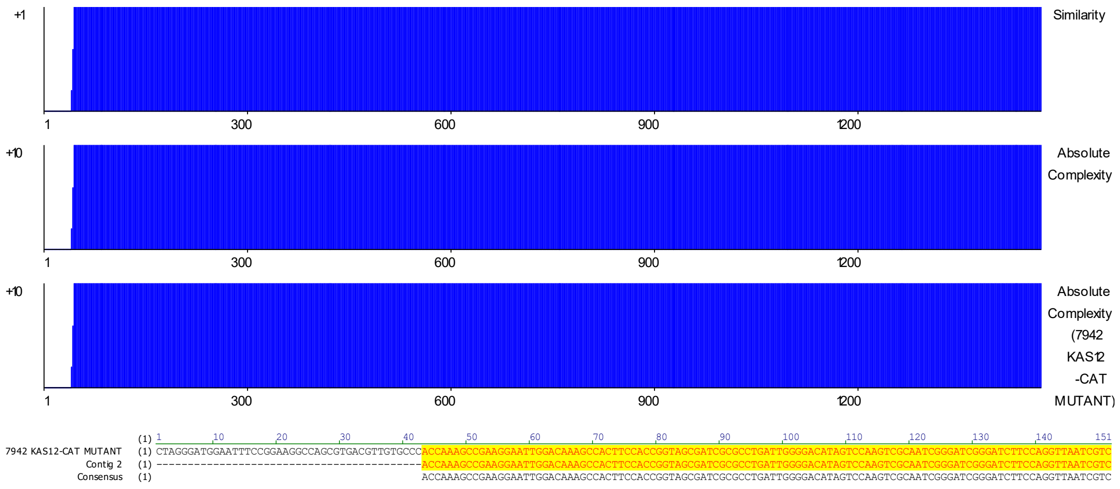

(b)

CTAGGGATGGAATTTCCGGAAGGCCAGCGTGACGTTGTGCCCACCAAAGCCGAAGGA
ATTGGACAAAGCCACTTCCACCGGTAGCGATCGCGCCTGATTGGGGACATAGTCCAAG
TCGCAATCGGGATCGGGATCTTCCAGGTTAATCGTCGGCGGCACCATGTCCTCAGCGAT
CGCGAGGGTTGCCGCTACCGCCTCAATTCCGCCGGAGCCCCCTAACAGGTGACCGGTC
ATCGACTTAGTCGAGCTGATCACGGTTTTGTAGGCGTGCTCACCTAGGGCTTTCTTAAT
AGCTGCCGTTTCGGTGCTGTCGTTGGCCGGTGTGCTGGTGCCGTGAGCATTGATGTAG
CTGACTTGGCTGGGCTGCAGATTGGCATCGCGGAGCGCCAACTCGATCGCCGCAACGG
GAATTTGAAGACAATAACTGCCTTAAAAAAATTACGCCCCGCCCTGCCACTCATCGCA
GTACTGTTGTAATTCATTAAGCATTCTGCCGACATGGAAGCCATCACAAACGGCATGAT
GAACCTGAATCGCCAGCGGCATCAGCACCTTGTCGCCTTGCGTATAATATTTGCCCATG
GTGAAAACGGGGGCGAAGAAGTTGTCCATATTGGCCACGTTTAAATCAAAACTGGTGA
AACTCACCCAGGGATTGGCTGAGACGAAAAACATATTCTCAATAAACCCTTTAGGGAA
ATAGGCCAGGTTTTCACCGTAACACGCCACATCTTGCGAATATATGTGTAGAAACTGCC
GGAAATCGTCGTGGTATTCACTCCAGAGCGATGAAAACGTTTCAGTTTGCTCATGGAA
AACGGTGTAACAAGGGTGAACACTATCCCATATCACCAGCTCACCGTCTTTCATTGCCA
TACGTAATTCCGGATGAGCATTCATCAGGCGGGCAAGAATGTGAATAAAGGCCGGATA
AAACTTGTGCTTATTTTTCTTTACGGTCTTTAAAAAGGCCGTAATATCCAGCTGAACGGT
CTGGTTATAGGTACATTGAGCAACTGACTGAAATGCCTCAAAATGTTCTTTACGATGCC
ATTGGGATATATCAACGGTGGTATATCCAGTGATTTTTTTCTCCATTTTAGCTTCCTTAGC
TCCTGCAGCGATCGGGGCCTTTTTCCAGCAAAACCGTCTGCTGGTCCTCCATCACCCTC
AAACCACCAATGCCTGAGCCGATCAGCACCCCGATCGCATCCGCATTCAGTTCAGTGA
TGTCCAGCTTGGCATCGGCGACTGCTTGGCGACTGGCAGCAACCGCCAGTTGTGCAAA
CCGATCCATCCGCTTAGCATCCTTGCGGTCCATGTACTGGGTGGGGTCAAAGTCCTTGA
CCTCCCCGGCAATTTTGCAGGCGTGACGAGACGCATCAAAGCCCCGAATCAGATCGAT
GCCGTTGCGACCGGCAAGGAGTCCCTGCCAATATTCCGTTGGATCATTACCGATGGGA
GTAATGGCTCCCAAACCAGTAATAACAACACGCTGGCGTCCGGTTTCAGTCAT

(c)

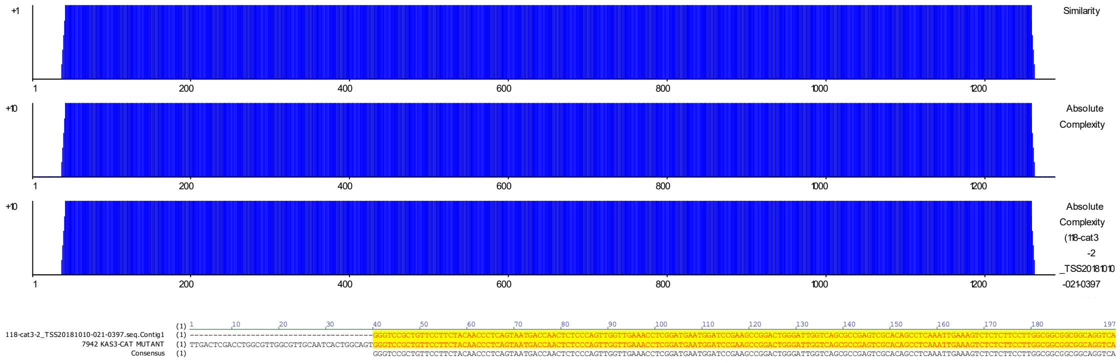

(d)

TTGACTCGACCTGGCGTTGGCGTTGCAATCACTGGCAGTGGGTCCGCTGTTCCTTCTAC
AACCCTCAGTAATGACCAACTCTCCCAGTTGGTTGAAACCTCGGATGAATGGATCCGA
AGCCGGACTGGGATTGGTCAGCGCCGAGTCGCACAGCCTCAAATTGAAAGTCTCTCTT
CCTTGGCGGCGGCGGCAGGTCAGTCAGCTCTCGAAGCTGCAGGGCTAGAAGCGACAT
CGGTTGATTTGATTTTGCTGGCGACGTCAACCCCCGACGATCTGTTCGGCAGTGCCTGC
CAAGTGCAAGGAGCTAAGGAAGCTAAAATGGAGAAAAAAATCACTGGATATACCACC
GTTGATATATCCCAATGGCATCGTAAAGAACATTTTGAGGCATTTCAGTCAGTTGCTCAA
TGTACCTATAACCAGACCGTTCAGCTGGATATTACGGCCTTTTTAAAGACCGTAAAGAA
AAATAAGCACAAGTTTTATCCGGCCTTTATTCACATTCTTGCCCGCCTGATGAATGCTCA
TCCGGAATTACGTATGGCAATGAAAGACGGTGAGCTGGTGATATGGGATAGTGTTCACC
CTTGTTACACCGTTTTCCATGAGCAAACTGAAACGTTTTCATCGCTCTGGAGTGAATAC
CACGACGATTTCCGGCAGTTTCTACACATATATTCGCAAGATGTGGCGTGTTACGGTGA
AAACCTGGCCTATTTCCCTAAAGGGTTTATTGAGAATATGTTTTTCGTCTCAGCCAATCC
CTGGGTGAGTTTCACCAGTTTTGATTTAAACGTGGCCAATATGGACAACTTCTTCGCCC
CCGTTTTCACCATGGGCAAATATTATACGCAAGGCGACAAGGTGCTGATGCCGCTGGCG
ATTCAGGTTCATCATGCCGTTTGTGATGGCTTCCATGTCGGCAGAATGCTTAATGAATTA
CAACAGTACTGCGATGAGTGGCAGGGCGGGGCGTAATTTTTTTAAGGCAGTTATTGTCT
TCAAATTCCCGTTGCATCCTCGAGAAGACGCTGTTCCACGCCGGGATCGATCGCCAAG
AAGTCGATTGGTTACTGCTACACCAGGCCAACCAACGCATTCTCGATGCAGTCGCCGA
TCGCCTTGATATTTCCCGTGATCGCGTACTTAGCAACTTGGTGAACTACGGCAATACCTC
CTCCGCCACGATCCCCTTGGTTTTGGATGAGGCCGTCAAAGCTGGGAAGATTCAATCG
GGCGACTTAATCGCTGCTTCAGGGTTTGGAGCAGGGTTGAGCTGGGGCGCAGCGCTGT
TCCGTTGGGGCACGGTGGTTTAG

Figure S3. DNA sequence analysis of the deletion mutants. (a) Alignment of construction of *fabB/F*-cat deletion mutation; (b) DNA fragment of *fabB/F*-cat; (c) Alignment of construction of *fabH*-cat deletion mutation; (d) DNA fragment of *fabH* -cat.
